# Versatile vector suite for the extracytoplasmic production and purification of heterologous His-tagged proteins in *Lactococcus lactis*

**DOI:** 10.1007/s00253-015-6778-8

**Published:** 2015-07-10

**Authors:** Jolanda Neef, Fin J. Milder, Danny G. A. M. Koedijk, Marindy Klaassens, Erik C. Heezius, Jos A. G. van Strijp, Andreas Otto, Dörte Becher, Jan Maarten van Dijl, Girbe Buist

**Affiliations:** Department of Medical Microbiology, University of Groningen, University Medical Center Groningen, Hanzeplein 1, P.O. Box 30001, 9700 RB Groningen, The Netherlands; Department of Medical Microbiology, University Medical Center Utrecht, PO G04.614, Heidelberglaan 100, 3584 CX Utrecht, The Netherlands; Institut für Mikrobiologie, Ernst-Moritz-Arndt Universität Greifswald, Friedrich-Ludwig-Jahn-Str. 15, D-17489 Greifswald, Germany

**Keywords:** *L. lactis*, Expression vectors, NICE, Usp45, Histidine tag, Secretion

## Abstract

Recent studies have shown that the Gram-positive bacterium *Lactococcus lactis* can be exploited for the expression of heterologous proteins; however, a versatile set of vectors suitable for inducible extracellular protein production and subsequent purification of the expressed proteins by immobilized metal affinity chromatography was so far lacking. Here we describe three novel vectors that, respectively, facilitate the nisin-inducible production of N- or C-terminally hexa-histidine (His_6_)-tagged proteins in *L. lactis*. One of these vectors also encodes a tobacco etch virus (TEV) protease cleavage site allowing removal of the N-terminal His_6_-tag from expressed proteins. Successful application of the developed vectors for protein expression, purification and/or functional studies is exemplified with six different cell wall-bound or secreted proteins from *Staphylococcus aureus*. The results show that secretory production of *S. aureus* proteins is affected by the position, N- or C-terminal, of the His_6_-tag. This seems to be due to an influence of the His_6_-tag on protein stability. Intriguingly, the *S. aureus* IsdB protein, which is phosphorylated in *S. aureus*, was also found to be phosphorylated when heterologously produced in *L. lactis*, albeit not on the same Tyr residue. This implies that this particular post-translational protein modification is to some extent conserved in *S. aureus* and *L. lactis*. Altogether, we are confident that the present vector set combined with the *L. lactis* expression host has the potential to become a very useful tool in optimization of the expression, purification and functional analysis of extracytoplasmic bacterial proteins.

**Electronic supplementary material:**

The online version of this article (doi:10.1007/s00253-015-6778-8) contains supplementary material, which is available to authorized users.

## Introduction

Over the past decades, a wide range of bacterial expression systems for heterologous protein production has been developed (Zerbs et al. 2009). Today, the Gram-negative bacterium *Escherichia coli* is one of the most commonly used organisms for large-scale heterologous protein production (Terpe 2006). This is due to the ease of handling, the multitude of available expression vectors and the relatively simple fermentation procedures for *E. coli* (Zerbs et al. 2009; Chen 2012). Despite these advantages, some clear disadvantages of the use of *E. coli* are evident. In the first place, *E. coli* is not capable of efficiently secreting heterologous proteins into the growth medium since exported proteins usually remain confined in the periplasm. Secondly, overexpression of heterologous proteins in *E. coli* often leads to the formation of high-density aggregates of misfolded proteins known as inclusion bodies. Thirdly, the post-translational modification of proteins that are heterologously produced in *E. coli* is likely to be different from the modification that these proteins undergo in their original host. Lastly, the inherent production of the well-known endotoxin lipopolysaccharide (LPS) is a major drawback for the clinical application of *E. coli*-derived recombinant proteins (Braun et al. 1999; Petsch and Anspach 2000; Sarvas et al. 2004; Westers et al. 2004; Neef et al. 2014).

While *E. coli* has become a preferred host for the cytoplasmic production of structurally simple biotherapeutics, other bacterial species, especially Gram-positive bacteria, are preferred hosts for the secretory production of structurally more challenging types of proteins. For example, *Bacillus* species are highly popular expression platforms for enzymes (Terpe 2006). Importantly, organisms such as *Bacillus subtilis* are generally regarded as safe (GRAS). Moreover, they can secrete proteins directly into the fermentation broth to high concentrations, thereby simplifying their downstream processing. However, bacilli often secrete endogenous proteases at high levels, which often requires the use of multiple protease-deficient strains (Li et al. 2004; Krishnappa et al. 2013). Alternatively, the Gram-positive bacterium *Lactococcus lactis* has been successfully applied for the secretory production of protease-sensitive proteins (Morello et al. 2008; Neef et al. 2014). This relates to the fact that this GRAS organism produces only two proteases that can potentially interfere with protein production. These two proteases, the cytoplasmic ClpP protease and the extracytoplasmic HtrA protease, are completely dispensable and their removal strongly reduces product degradation (Morello et al. 2008; Poquet et al. 2000; Miyoshi et al. 2002; Cortes-Perez et al. 2006). Moreover, the unwanted autolysis of *L. lactis* cells is prevented by the removal of the major autolysin AcmA which, combined with an *htrA* deletion, leads to the stable and efficient production of secreted proteinaceous antigens of *Staphylococcus aureus* (Neef et al. 2014).

Several inducible expression systems have been developed for *L. lactis* (Morello et al. 2008) of which the nisin-inducible (NICE) system is the most efficient and extensively used (Mierau 2005). This system is based on the regulation of the *nisA* promoter by the food-grade lantibiotic nisin, which activates the NisRK two-component regulatory system (De Ruyter et al. 1996). The NICE system has thus been used for production of a wide range of homologous and heterologous proteins, including vaccines (Zhou et al. 2006).

The purification of overproduced proteins can be facilitated by particular tags that bind with high affinity to a specific matrix. The hexa-histidine (His_6_)-tag is the most widely used tag and ensures efficient separation by metal affinity chromatography (Jones et al. 1995). However, the exact placement of these tags can influence the solubility and/or stability of overproduced proteins (Woestenenk et al. 2004). To circumvent the latter problems, changing the location of the His_6_-tag from the N- to the C-terminus or vice versa may prove beneficial. Notably, although the His_6_-tag has usually limited impact on protein structure or function (Terpe 2003), it is desirable to remove it prior to structure-function studies (Arnau et al. 2011). Therefore, a specific protease cleavage site, e.g. for the tobacco etch virus (TEV) protease, is often placed between the target protein and the affinity tag.

In this study, we describe an expression vector set that facilitates convenient exploration of nisin-inducible protein production in *L. lactis*. As shown with a representative panel of extracytoplasmic proteinaceous antigens from *S. aureus*, the overproduced proteins can be purified by metal affinity chromatography using N- or C-terminal His_6_-tags. The latter is useful since our present results show that, also in *L. lactis*, the exact position of the His_6_-tag affects production efficiency and/or protein stability. In one vector configuration, the His_6_-tag can be removed from the expressed proteins by cleavage with the TEV protease. Of note, we show that *L. lactis* is capable of phosphorylating the IsdB protein of *S. aureus*.

## Materials and methods

### Bacterial strains and growth conditions

Strains and plasmids are listed in Table 1. *E. coli* was grown at 37 °C in Lysogeny broth (LB; Becton Dickinson, Breda, The Netherlands) with ampicillin (100 μg/ml) for plasmid selection. *L. lactis* was grown at 30 °C in M17 broth (Oxoid Limited, Hampshire, UK) supplemented with 0.5 % glucose (*w*/*v*) (GM17). Chloramphenicol (5 μg/ml) was added when needed. For nisin production, the *L. lactis* NZ9700 strain was cultured in GM17 and the cell-free supernatant was used for induction of the P_nisA_ promoter in a 1:1000 dilution at OD_600_ ~ 0.5 (Kuipers et al. 1997).

**Table 1:** Bacterial strains and plasmids used in this study

Strain or plasmid	Relevant phenotype(s) or genotype(s)	Source and reference
Strains		
*L. lactis* NZ9700	Nisin producer	NIZO culture collection, Ede, The Netherlands (Kuipers et al. 1993)
*L. lactis* PA1001	MG1363 *pepN*::*nisRK*, allows nisin-inducible expression, Δ*acmA* Δ*htrA*	Mucosis culture collection, Groningen, The Netherlands (Bosma et al. 2006)
*S. aureus* Newman	NCTC 8178 clinical isolate	PHE culture collection NCTC8178 (Duthie and Lorenz 1952)
*S. aureus* USA300	Community-acquired MRSA isolate	ATCC strain BAA-1717 (McDougal et al. 2003)
*S. aureus* N315	Hospital-acquired MRSA	(Kuroda et al. 2001)
*S. aureus* NCTC8325	Restriction-deficient derivative of NCTC 8325; cured of all known prophages	WDCM culture collection 154 (Kreiswirth et al. 1983)
Plasmids		
pET302/NT	Ap^R^, containing P_T7_, *lacO*, MCS, *his*_*6*_, T7 terminator	Life Technologies
pEF110	pET302/NT derivative, Ap^R^, containing P_T7_, *his*_*6*_, *Bam*HI/*Eco*RI-*Xba*I/*Not*I cloning sites	Laboratory collection J.A.G. van Strijp
pEF111	pET302/NT derivative, Ap^R^, containing P_T7_, *his*_*6*_, TEV cleavage site, *Bam*HI/*Eco*RI-*Xba*I/*Not*I cloning sites	Laboratory collection J.A.G. van Strijp
pEF210	pET302/NT derivative, Ap^R^, containing P_T7_, *Bam*HI/*Eco*RI-*Xba*I/*Not*I cloning sites, *his*_*6*_	Laboratory collection J.A.G. van Strijp
pNG400	Cm^R^, containing P_*nisA*_, SS_*usp45*_, and a transcription terminator	(Bosma et al. 2006)
pNG4110	pNG400 derivative, containing *his*_*6*_, *Bam*HI/*Eco*RI-*Xba*I/*Not*I cloning sites	This study
pNG4111	pNG400 derivative, containing *his*_*6*_, TEV cleavage site, *Bam*HI/*Eco*RI-*Xba*I/*Not*I cloning sites	This study
pNG4210	pNG400 derivative, containing *Bam*HI/*Eco*RI-*Xba*I/*Not*I cloning sites, *his*_*6*_ followed by a Stop codon	This study

*Ap* ^*R*^ ampicillin resistance gene, *Cm* ^*R*^ chloramphenicol resistance gene, *P*_*T7*_ IPTG inducible T7-promoter, *P*_*nisA*_ nisin-inducible promoter, *his*_*6*_ 6 histidine tag, *SS*_*usp45*_ signal sequence of *usp45*, *MCS* multiple cloning site

### General molecular biology

Enzymes and buffers were from New England Biolabs (Ipswich, UK) and Fermentas (Landsmeer, The Netherlands). PCR was performed using a Bio-Rad C1000 Thermal Cycler (Richmond, CA). Primers were from Eurogentec (Maastricht, The Netherlands) (Table 2 and Table S1). The polymerases PFU (Fermentas, Landsmeer, The Netherlands), Pwo (Roche, Woerden, The Netherlands) and Taq (Life Technologies, Bleiswijk, The Netherlands) were used according to the manufacturer. PCR products were purified using the High Pure PCR Purification Kit from Roche (Woerden, The Netherlands). Plasmid purification was performed using the Plasmid Isolation Kit from Analytik Jena AG (Jena, Germany); *L. lactis* was lysed by incubation with lysozyme (4 mg/ml; Sigma-Aldrich, Zwijndrecht, The Netherlands) for 10 min at 55 °C in resuspension buffer followed by addition of lysis buffer. *L. lactis* was transformed by electroporation using a Gene Pulser (Biorad; Leenhouts and Venema 1993). Nucleotide sequence analysis was performed by Eurofins DNA (Ebersberg, Germany).

**Table 2:** Primers used for the construction of the expression vectors

Primer	5′ → 3′ nucleotide sequence^a^	Restriction site^b^
NHis_F	TATGCACCATCACCATCACCATGGATCCGAATTCCTCGAGGCGGCCGCATAAA	
NHis_R	ACGTGGTAGTGGTAGTGGTACCTAGGCTTAAGGAGCTCCGCCGGCGTATTTCTAG	
CHis_F	TATGGGATCCGAATTCCTCGAGGCGGCCGCACACCATCACCATCACCATTAAA	
CHis_R	ACCCTAGGCTTAAGGAGCTCCGCCGGCGTGTGGTAGTGGTAGTGGTAATTTCTAG	
NHisTEV_F	TATGCACCATCACCATCACCATGAGAACCTGTACTTCCAGGGATCCGAATTCCTCGAGGCGGCCGCATAAA	
NHisTEV_R	ACGTGGTAGTGGTAGTGGTACTCTTGGACATGAAGGTCCCTAGGCTTAAGGAGCTCCGCCGGCGTATTTCTAG	
pEF110.f	CCCCGTCTC *CCATGCA*CCATCACCATCATCATGGATCCGAA	B*sm*BI
pEF110.r	CCCAAGCTT **TTA** CCATGGTGCGGCCGCCTCGAGG	*Hin*dIII and *Not*I
pEF111.f	CCCCGTCTC *CCATGCA*CCATCACCATCACCATGAG	*Bsm*BI
pEF111.r	CCCAAGCTT **TTA**TGCGGCCGCCTCGAGG	*Hin*dIII
pEF210.f	CCCCCATGGGATCCGAATTCCTCGAGGC	*Nco*I
pEF210.r	CCCAAGCTT **TTA**ATGGTGATGGTGATGGTGTGC	*Hin*dIII

^a^Restriction site underlined, stop codon in bold

^b^ *Bsm*BI restriction resulting in *Nco*I overhang in italic

### Construction of expression vectors

**Fig. 1:**
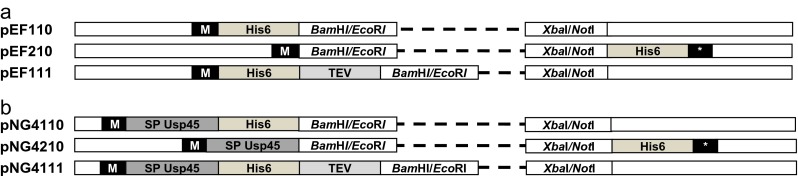
Expression cassettes present in the vectors for heterologous protein production in *E. coli* or *L. lactis*. **a** Expression cassettes of the *E. coli* pEF vectors, encoding an N-terminal His_6_-tag (pEF110), a C-terminal His_6_-tag (pEF210) or a TEV-removable (TEV) N-terminal His_6_-tag (pEF111). Restriction sites for cloning (*Bam*HI/*Eco*RI or *Xba*I/*Not*I, respectively), start codons (M) and stop codons (*) are indicated. **b** Expression cassettes of the *L. lactis* pNG vectors, encoding an N-terminal His_6_-tag (pNG4110), a C-terminal His_6_-tag (pNG4210) or a TEV-removable (TEV) N-terminal His_6_-tag (pNG4111). Restriction sites for cloning (*Bam*HI/*Eco*RI or *Xba*I/*Not*I, respectively), start codons (M), the Usp 45 signal sequence (SP Usp45) and stop codons (*) are indicated

The *E. coli* pEF vector set is based on plasmid pET302/NT. A total of three primer pairs were created (NHis_F/NHis_R for pEF110, CHis_F/CHis_R for pEF210, NHisTEV_F/NHisTEV_R for pEF111) (Table 2) containing an *Nde*I and *Bgl*II overhang at their 5′ and 3′ ends. These primers were annealed by incubation at a 1:1 ratio at 94 °C and stepwise-controlled temperature drops to room temperature. The fragments were ligated into the *Nde*I and *Bam*HI digested pET302/NT vector resulting in deletion of the original vector-derived *Bam*HI site and introduction of a multiple cloning site comprising *Bam*HI, *Eco*RI, *Xho*I and *Not*I restriction sites (Fig. 1). Following transformation in *E. coli* TOP10F cells, the prepared constructs were sequence-verified.

*L. lactis* vectors were constructed based on the pEF vectors by amplifying the multiple cloning site together with the His_6_-tag and the TEV cleavage site (encoded by the specific amino acid sequence EQLYFOG (Arnau et al. 2011) using the primers in Table 2. For construction of pNG4110 and pNG4111, the primer combinations pEF110.fw/pEF110.rev and pEF111.fw/pEF111.rev were used with plasmids pEF110 and pEF111 as templates, respectively. PCR products were digested using *Hin*dIII and *Bsm*BI resulting in *Hin*dIII and *Nco*I overhangs and ligated into plasmid pNG400 digested with *Hin*dIII and *Nco*I. For the construction of pNG4210, primers pEF210.fw/pEF210.rev were used with plasmid pEF210 as template DNA. The PCR products and receiving plasmid pNG400 were digested using *Nco*I and *Hin*dIII. After ligation, the resulting vectors were used to transform *L. lactis* PA1001. For expression of extracellular proteins of *S. aureus* in *L. lactis*, the respective genes were amplified by PCR with Pwo polymerase from chromosomal DNA of *S. aureus* strains USA300 or NCTC8325 (see Table 3). Genes amplified with the f1/r2 primer sets were cloned in pNG4110 and pNG4111, and genes amplified with the f1/r1 primer sets in pNG4210 using the *BamH*I/*Not*I restriction sites (Table S1). Ligation mixtures were introduced into *L. lactis* PA1001. The resulting vectors were sequence-verified.

**Table 3:** *S. aureus* proteins used in this study

Name and function	NCBI^a^	Aa^b^	pI^c^	kDa^d^	Constructs
SA0620, Secretory antigen SsaA homologue (*ssaA* like)	USA300HOU_0686	26-265	6.44	27.2	pNG4110-*sa0620*
			6.26	28.0	pNG4111-*sa0620*
			6.44	27.1	pNG4210-*sa0620*
FtsL, cell division	USA300HOU_1120	66-133	9.41	8.9	pNG4110-*ftsL*
			9.02	9.7	pNG4111-*ftsL*
			9.41	9.1	pNG4210-*ftsL*
ClfB, clumping factor B	USA300HOU_2630	45-567	4.68	57.8	pNG4110-*clfB*
			4.65	58.6	pNG4111-*clfB*
			4.68	57.9	pNG4210-*clfB*
Hypothetical protein SA2100, similar to autolysin E	USA300HOU_2288	27-258	8.95	27.7	pNG4110-*sa2100*
			8.62	28.5	pNG4111-*sa2100*
			8.95	27.9	pNG4210-*sa2100*
Pro-Atl, pro-peptide autolysin Atl	SAOUHSC_00994	29-199	9.02	19.0	pNG4110-*pro*-*atl*
			7.87	19.8	pNG4111-*pro*-*atl*
			9.02	18.9	pNG4210-*pro*-*atl*
IsdB, iron-regulated heme-iron binding protein		41-609	9.18	64.9	pNG4110-*isdB*
			9.10	65.7	pNG4111-*isdB*
			9.18	65.2	pNG4210-*isdB*

^a^NCBI genome annotation of *S. aureus* strain USA300_TCH1516 (SAUSA300) or NCTC8325 (SAOUHSC)

^b^Numbers of first and last amino acid residues of the proteins expressed in *L. lactis*

^c^Isoelectric point

^d^Molecular weight

### Lithium dodecyl sulphate-polyacrylamide gel electrophoresis and Western blotting

For lithium dodecyl sulphate-polyacrylamide gel electrophoresis (LDS-PAGE), cells were resuspended in LDS buffer (Life Technologies) and disrupted by bead beating with 0.1 μm glass beads (Biospec Products, Bartlesville, USA) using a Precellys24 (Bertin Technologies, Montigny-le-Bretonneux, France), while secreted proteins in the culture medium were precipitated with 10 % trichloroacetic acid (TCA). Protein samples were incubated for 10 min at 95 °C, separated by LDS-PAGE using 10 % NuPAGE gels (Invitrogen) and stained with SimplyBlue^TM^ SafeStain (Life Technologies). For Western blotting, proteins were transferred to a nitrocellulose membrane (Protran®, Schleicher & Schuell, Dassel, Germany). Immunodetection was performed using anti-His-tag antibodies (Life Technologies). Bound antibodies were visualized using fluorescently labeled secondary antibodies (IRDye 800 CW from LiCor Biosciences, NE, USA). Membranes were scanned for fluorescence at 800 nm using the Odyssey Infrared Imaging System (LiCor Biosciences).

### Protein production and isolation

Overnight cultures of *L. lactis* were diluted 1:20 in GM17 medium containing chloramphenicol. Induction of P_nisA_ with nisin was performed for 16 h. Cells producing *S. aureus* protein SA0620 were resuspended in binding buffer (20 mM sodium phosphate, pH 7.4, 0.5 mM NaCl, 50 mM imidazole) and disrupted by bead beating. The *S. aureus* proteins SA2100 and pro-Atl were precipitated from the growth medium of *L. lactis* with 10 % TCA. The SA0620 protein in cell-free extracts and the TCA-precipitated SA2100 and pro-Atl proteins were purified by metal affinity chromatography with His Mag SepharoseTM Ni beads (Mag beads; GE Healthcare, Little Chalfont, UK). Incubation with Mag beads was performed for ~1 h at room temperature; unbound proteins were removed by washing with binding buffer, and bound proteins were eluted with elution buffer (20 mM sodium phosphate, pH 7.4, 0.5 M NaCl and 500 mM imidazole).

### Protein activity assays

To analyse the possibility of the TEV cleavage, *L. lactis* culture medium containing His_6_-TEV-FtsL, was dialysed against phosphate buffered saline (PBS) and incubated 16 h at 4 °C with 10 U TurboTEV protease (Eton Bioscience, Inc., San Diego, CA). Proteins were separated on a 10 % NuPAGE gel and stained with SimplyBlue.

Cell wall hydrolase activity of His_6_-tagged derivatives of the SA2100 protein was analysed in zymograms using sodium dodecyl sulfate-polyacrylamide (SDS-PAA) gels (12.5 %) containing 0.15 % autoclaved, lyophilized *Micrococcus lysodeikticus* ATCC 4698 cells (Sigma-Aldrich.), as described (Buist et al. 1997).

Clumping activity of ClfB was analysed by growing induced PA1001 cells expressing *clfB* in a 12-well microtitre plate. After overnight induction with nisin, the plate was gently stirred. Cell clumping was visualized using a G-Box Chemi XT16 (Syngene, Cambridge, UK).

### Analysis of phosphorylation

Phosphorylation of His_6_-IsdB and His_6_-TEV-IsdB was visualized by LDS-PAGE and subsequent staining with Pro-Q® Diamond phosphoprotein gel stain (Life Technologies). Cytosolic cell fractions were produced by bead beating of overnight nisin-induced cells. Cell debris was removed by centrifugation. Prior to separation by LDS-PAGE, cytosolic and secreted proteins were delipidated and desalted according to the instructions for use of the Pro-Q stain. Protein staining was visualized with the G-Box.

Gel pieces containing putatively phosphorylated proteins were prepared for mass spectrometric (MS) analysis as described (Bonn et al. 2014). Peptides were eluted and subjected to high-resolution and high mass accuracy MS measurements on an Orbitrap Elite coupled online to a Proxeon EASY-nLC 1000. The Orbitrap Elite was operated in data-dependent MS/MS mode at a resolution of *R* = 60,000 in the MS1 with the lockmass option enabled. Data-dependent triggering of fragment scans was set on the 20 most intense precursor ions. Multistage activation (MSA) was used for enhanced fragmentation of putative phosphate group containing ions as described (Basell et al. 2014). MS/MS spectra were searched against a target-decoy database including all protein sequences of *S. aureus* USA300 extracted from the UniProt database in addition to common laboratory contaminants and an appended set of the reversed sequences. Database searching was performed by Sequest (Thermo Fisher Scientific, San Jose, CA, USA; version v.27, rev. 11). Peptide hits were filtered with Scaffold (version Scaffold_4.3.4, Proteome Software Inc., Portland, OR). Mass tolerance for peptide identification on MS and MS/MS peaks were 10 ppm and 1 Da, respectively. Up to two missed tryptic cleavages were allowed. Methionine oxidation and cysteine carbamidomethylation, as well as phosphorylation at serine, threonine or tyrosine, were set as variable modifications. Peptide identifications were accepted if they matched the following criteria: deltaCn scores of greater than 0.10 and XCorr scores of greater than, 2.5, 3.5 and 3.5 for doubly, triply and quadruply charged peptides. Protein identifications were accepted if they contained at least two identified peptides. Proteins that contained similar peptides and could not be differentiated based on MS/MS analysis alone were grouped to satisfy the principles of parsimony.

## Results

### Development of a pNG vector set for heterologous protein production in *L. lactis*

In previous studies, the pEF vector set was successfully used for heterologous protein expression in *E. coli* (Bardoel et al. 2012; Pel et al. 2014). This vector set offered convenient possibilities for N- or C-terminal His_6_-tagging of expressed proteins (pEF110 and pEF210, respectively) and for TEV-mediated tag removal (pEF111; Fig. 1a). We therefore implemented the respective cloning sites, His_6_-tag sequences and TEV cleavage site sequence in the pNG series of *L. lactis* expression vectors. In addition, the pNG vectors were provided with the *nisA* promoter and the signal sequence of Usp45 for nisin-inducible secretory protein production. Specifically, this resulted in pNG4110 for expression of N-terminally His_6_-tagged proteins, pNG4210 for expression of C-terminally His_6_-tagged proteins and pNG4111 for expression of proteins with a TEV protease-removable N-terminal His_6_-tag (Fig. 1b).

The versatility of the pNG vectors was tested by expressing six different extracytoplasmic proteins of *S. aureus*. Notably, these included a secreted protein (SA0620), a membrane-associated protein (FtsL), two covalently cell wall-bound proteins (ClfB and IsdB), a non-covalently cell wall-bound protein (SA2100) and the pro-peptide of the major autolysin Atl (Table 2). To express these proteins, the *L. lactis* strain PA1001 was used, which has an improved stability due to a deletion of the *acmA* gene and a reduced proteolytic activity due to deletion of the *htrA* gene (Neef et al. 2014).

### Position of the His_6_-tagging affects SA0620 production in *L. lactis*

**Fig. 2:**
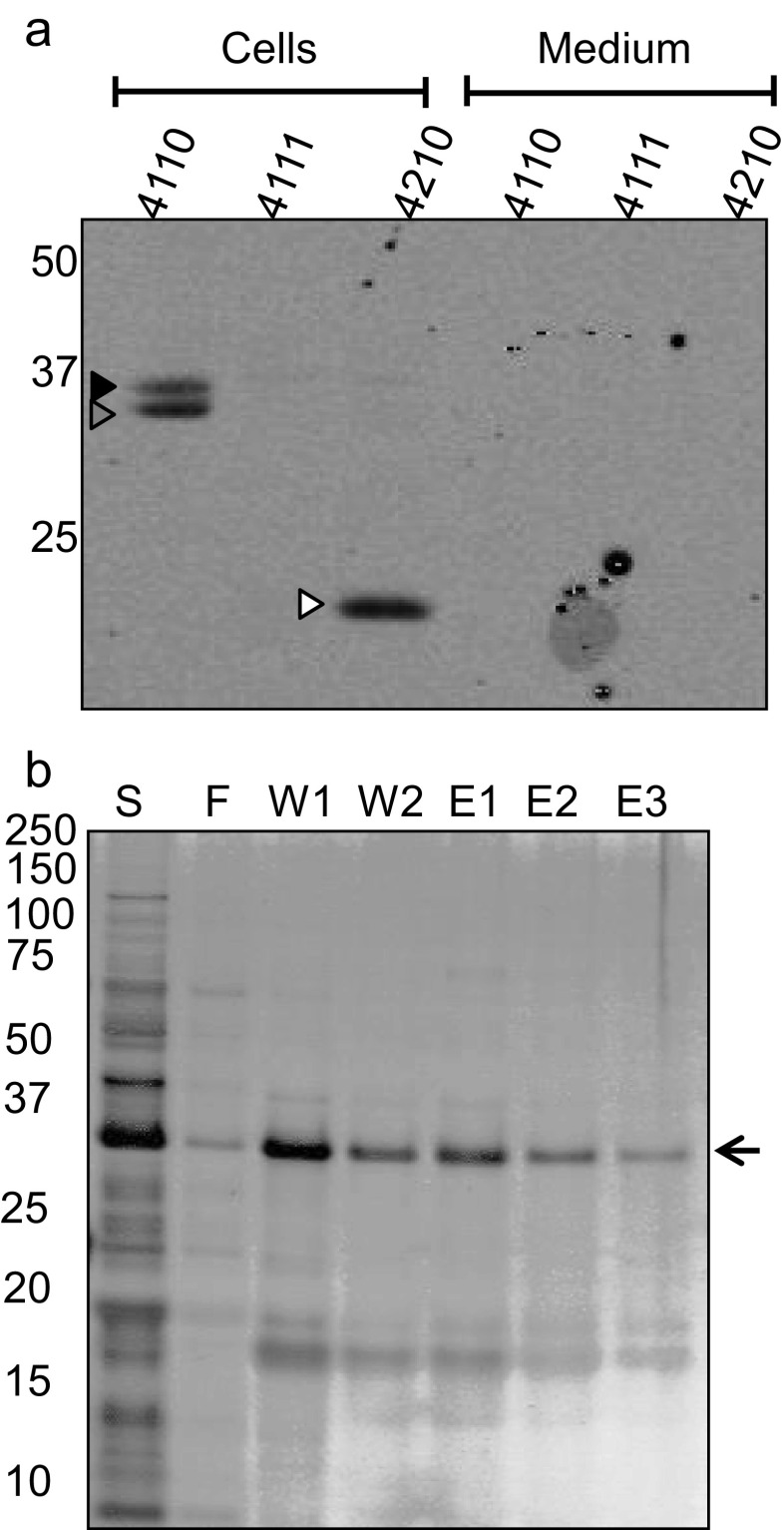
Production and purification of the *S. aureus* SA0620 protein. **a** Cell lysates (Cells) and TCA-precipitated growth medium (Medium) of *L. lactis* PA1001 expressing His_6_-SA0620 (4110), His_6_-TEV-SA0620 (4111) or SA0620-His_6_ (4210) were analysed by Western blotting using anti-His_6_ antibodies. The *black arrowhead* indicates a potential precursor form of SA0620, the *grey arrowhead* indicates the mature-sized SA0620 protein and the *white arrowhead* indicates a degradation product. **b** The His_6_-SA0620 (4110) was purified form the disrupted cells in the presence of 6 M urea. The start material (S), flow-through fraction (F), wash fractions (W) and elution fractions (E) were analysed by LDS-PAGE and separated proteins were detected by silver staining. The position of His_6_-SA0620 is indicated by an *arrow* and a co-purified degradation product of this protein is marked (*). The positions of Mw marker proteins are indicated

In a previous study, we observed that the secretory antigen SsaA homologue SA0620 was not produced in *L. lactis* PA1001 when expressed with a C-terminal His_6_-tag from plasmid pNG400 (Neef et al. 2014). To test whether this might be due to the location of the His_6_-tag, the gene for SA0620 was cloned in plasmids pNG4110, pNG4111 and pNG4210. After overnight induction with nisin, the expression of the resulting His_6_-tagged proteins was analysed by LDS-PAGE and Western blotting. This showed that effective expression of the full-length SA0620 precursor and mature proteins (30.4 and 27.7 kDa, respectively) was only achieved when this protein was synthesized with an N-terminal His_6_-tag as provided by pNG4110 (Fig. 2a). Precursor forms of SA0620 were observed in low amounts when expressed from pNG4111 or pNG4210, and in the latter case, also a degradation product of approximately 17 kDa was detectable (Fig. 2a). For the His_6_-TEV-SA0620 variant produced from pNG4111, not even a degradation product was detectable. Notably, despite the fusion of SA0620 to the signal peptide of Usp45, no SA0620 or fragments thereof were detectable in the growth medium (Fig. 2a). Nevertheless, the expression in pNG4110 did allow purification of the mature SA0620 protein and a degradation product from cells disrupted by bead beating and subsequent treatment with 6 M urea, as illustrated in Fig. 2b. Together, these data show that the N-terminal location of the His_6_-tag is critical for successful production and subsequent isolation of the full-length *S. aureus* antigen SA0620.

**Fig. 3:**
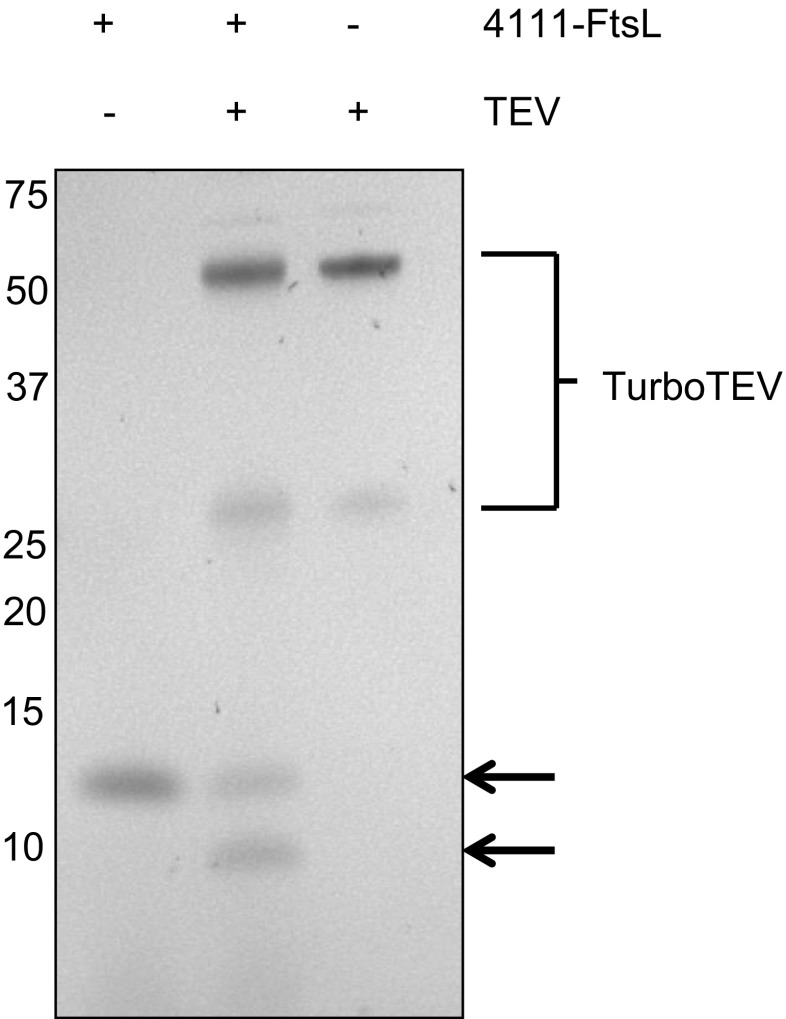
TEV cleavage of His_6_-FtsL. Production of the His_6_-TEV-FtsL protein by *L. lactis* PA1001 pNG4111-*ftsL* was induced overnight with nisin. Growth medium containing His_6_-TEV-FtsL was dialysed against PBS and then the TurboTEV protease (52 kDa) was added. Fractions with or without TurboTEV were separated by LDS-PAGE and stained with SimplyBlue. The positions of Mw marker proteins are indicated

### TEV protease-mediated His_6_-tag removal from the secreted His_6_-TEV-FtsL protein

To determine whether the His_6_-tag can proteolytically be removed from proteins expressed from the pNG4111 vector, by means of the encoded TEV cleavage site, the *S. aureus* cell division membrane protein FtsL was used. Specifically, the *ftsL* gene was expressed lacking the transmembrane helix. After induction with nisin, the His_6_-TEV-FtsL fusion protein was efficiently secreted into the culture medium of *L. lactis* (Fig. 3). Upon removal of cells by centrifugation, dialysis against PBS and incubation with the TurboTEV protease, cleavage of His_6_-TEV-FtsL was analysed by LDS-PAGE and SimplyBlue staining. In the samples incubated in the absence of TurboTEV protease, the presumably complete fusion protein was detectable upon LDS-PAGE and SimplyBlue staining (Fig. 3). Of note, the apparent molecular weight (Mw) of the fusion protein judged by its mobility on LDS-PAGE was ~12 kDa, while the predicted Mw is only 9.5 kDa. Upon incubation in the presence of the TEV protease, an additional protein band with an apparent Mw of ~10 kDa was detectable. This is in agreement with the predicted mass reduction of the His_6_-TEV-FtsL fusion by ~1.7 kDa upon cleavage at the TEV site. The cleavage product was not detected in the control sample, solely TurboTEV protease, indicating the product is His_6_-TEV-FtsL derived (Fig. 3). Together, these findings show that efficient extracellular protein production can be achieved with pNG4111 and that the His_6_-tag can be removed from the secreted fusion product by cleavage with the TEV protease.

**Fig. 4:**
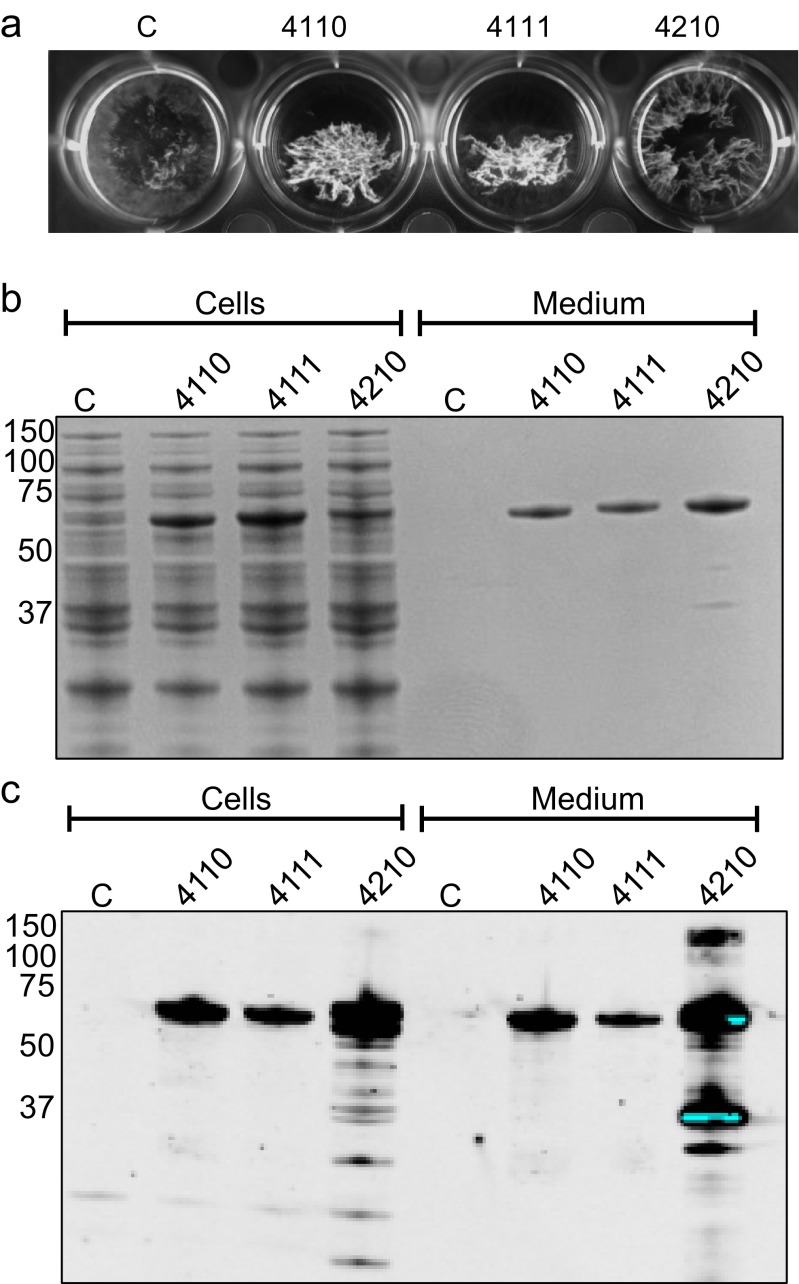
Functional expression of the *S. aureus* ClfB protein in *L. lactis*. **a** Clumping of *L. lactis* PA1001 cells producing His_6_-ClfB (4110), His_6_-TEV-ClfB (4111) or ClfB-His_6_ (4210) upon overnight induction with nisin. No clumping was observed in the absence of nisin, as shown under (C) for a non-induced control culture of *L. lactis* PA1001 harbouring pNG4110-*clfB*. **b** and **c** Cell and growth medium fractions of *L. lactis* PA1001 producing His_6_-ClfB (4110), His_6_-TEV-ClfB (4111) or ClfB-His_6_ (4210) were analysed by LDS-PAGE. Gels were either stained with SimplyBlue SafeStain (**b**) or used for Western blotting (**c**) with anti-histidine antibodies. As a control, non-induced *L. lactis* PA1001 pNG4110-*clfB* was included in the analysis (C). The positions of Mw marker proteins are indicated

### Functional expression of ClfB in *L. lactis*

To assess whether pNG4110, pNG4111 and pNG4210 facilitate the expression of proteinaceous antigens in *L. lactis* that are covalently bound to the cell surface of *S. aureus*, the ClfB protein was used. Specifically, the *clfB* gene was introduced into the pNG expression vectors without the 3′ sequences that encode the LPxTG motif for sortase recognition and cleavage and the C-terminal transmembrane domain. Furthermore, the original signal peptide of ClfB was replaced with the Usp45 signal peptide. Remarkably, clumping of ClfB-expressing *L. lactis* strains was detectable (Fig. 4a) in cultures upon overnight induction with nisin. Interestingly, this clumping phenotype was not observed for control cells not expressing ClfB, which suggests that ClfB lacking the LPxTG motif still associates to the cell surface. To verify ClfB production and possible secretion, the cells were separated from the growth medium and both fractions were analysed by LDS-PAGE. Gels were then either stained with SimplyBlue (Fig. 4b) or used for Western blotting and immunodetection using anti-His_6_ antibodies (Fig. 4c). His_6_-tagged proteins were detectable both in the cellular and growth medium fractions (Fig. 4b, c) indicating ClfB was efficiently expressed from all three vectors (pNG4110, pNG4111 and pNG4210). Notably, several degradation products of the C-terminally His_6_-tagged ClfB were detectable in the cell and growth medium fractions, while this was not the case for the N-terminally His_6_-tagged forms of ClfB. This suggests that C-terminal His_6_-tagging makes ClfB more prone to degradation by as yet unknown proteases of *L. lactis*. Furthermore, the results shown in Fig. 4 suggest that the location of the His_6_-tag may influence ClfB-mediated clumping of *L. lactis* cells, since cells expressing ClfB-His_6_ from pNG4210 showed a milder clumping phenotype than cells expressing His_6_-ClfB or His_6_-TEV-Clfb from pNG4110 or pNG4111, respectively. Whether this relates to differences in the produced amounts of His_6_-tagged ClfB is difficult to say since the position of the His_6_-tag seems to influence either the efficiency of SimplyBlue protein staining or of immunodetection with His_6_-specific antibodies.

### Peptidoglycan cleavage activity of SA2100

**Fig. 5:**
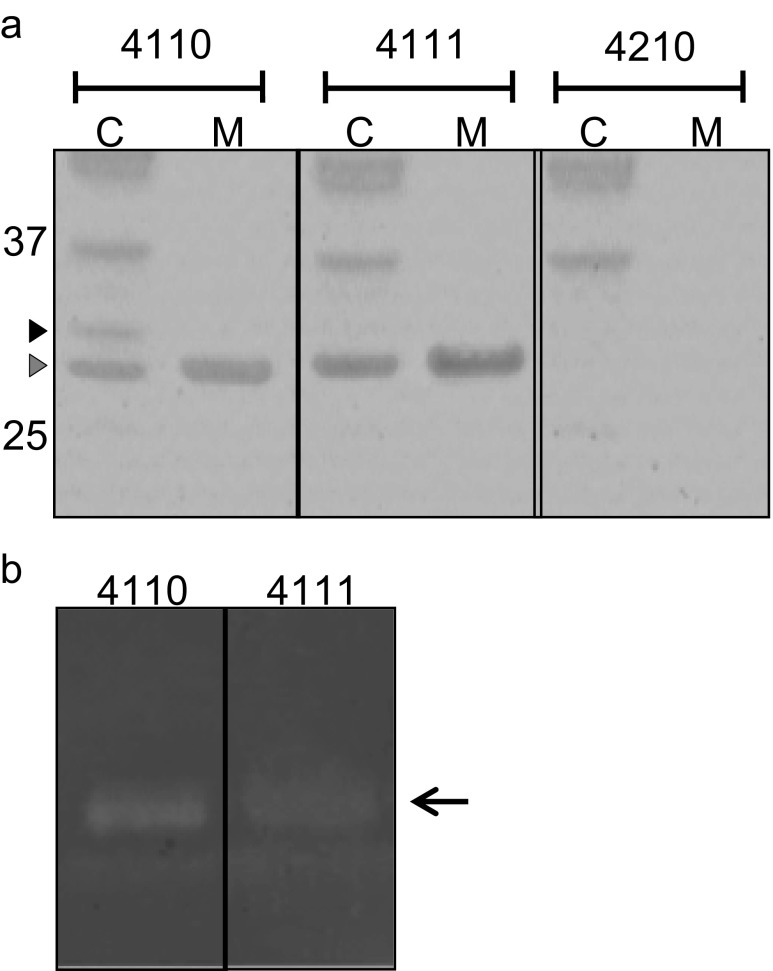
Functional expression of the *S. aureus* SA2100 protein in *L. lactis*. **a** Cells (C) and growth medium (M) fractions of *L. lactis* PA1001 producing His_6_-SA2100 (4110), His_6_-TEV-SA2100 (4111) or SA2100-His_6_ (4210) were analysed by LDS-PAGE and stained with SimplyBlue SafeStain. The *black arrowhead* indicates a potential precursor form of His_6_-SA2100, and the *grey arrowhead* indicates matured SA2100. The positions of Mw marker proteins are indicated. **b** The cell wall hydrolyzing activity of His_6_-SA2100 (4110) and His_6_-TEV-SA2100 (4111) was analysed by zymography upon SDS-PAGE in gels containing *M. lysodeikticus* cell wall extract. Upon electrophoresis and renaturation of separated proteins, the gel was stained with methylene blue as described in the “Materials and methods” section. A zone of cell wall-degrading activity, which corresponds to the position of mature SA2100 in the gel upon electrophoresis is indicated with an *arrow*

To investigate whether non-covalently cell wall-bound proteins of *S. aureus* can be produced in *L. lactis* using pNG4110, pNG4111 and/or pNG4210, the gene encoding the *S. aureus* SA2100 protein was cloned in these vectors. In the process, the native signal sequence of SA2100 was replaced with the Usp45 signal sequence. As shown in Fig. 5, cell-associated and secreted mature forms of SA2100 were only detectable by expression from pNG4110 or pNG4111, i.e. with N-terminal His_6_-tags, and not when expressed from the pNG4210 vector that encodes the C-terminally His_6_-tagged protein. Furthermore, a precursor from of His_6_-SA2100 was detectable in cells expressing this protein from pNG4110. Together, these observations support the view that the location of the His_6_-tag can be critical for effective protein production in *L. lactis*.

Notably, SA2100 is homologous to the autolysin E protein of *S. aureus* and contains a C-terminal domain with similarity to the so-called lysozyme subfamily 2 (LYZ2, smart00047; see the CCD database of NCBI at http://www.ncbi.nlm.nih.gov/Structure/cdd/cdd.shtml). The latter protein family has a peptidoglycan-hydrolyzing activity that is comparable to muramidase activity (Buist et al. 1995). To investigate whether the heterologously expressed and secreted form of SA2100 displays such an activity, a zymogram assay for the degradation of cell wall fragments from *M. lysodeikticus* was employed. As shown by zymographic analysis, the purified His_6_-SA2100 and His_6_-TEV-SA2100 were both capable of degrading cell wall fragments of *M. lysodeikticus* (Fig. 5b), which demonstrates that SA2100 is indeed a cell wall-degrading enzyme.

### Expression of the Atl pro-peptide in *L. lactis*

**Fig. 6:**
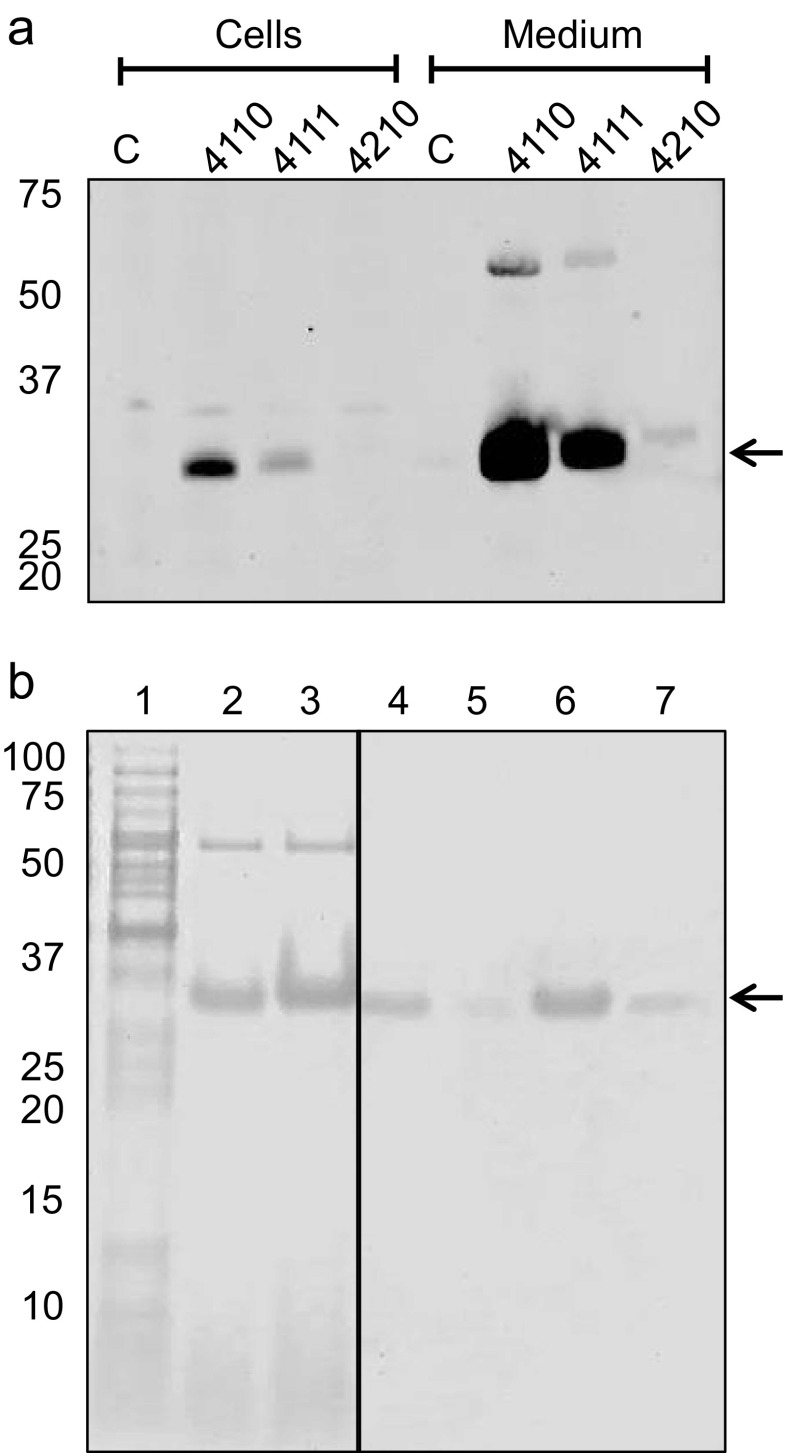
Production and purification of the *S. aureus* pro-Atl pro-peptide. **a** Cells and growth medium fractions of *L. lactis* PA1001 producing His_6_-Pro-Atl (4110), His_6_-TEV-Pro-Atl (4111) or Pro-Atl-His_6_ (4210) were analysed by LDS-PAGE and subsequent Western blotting using anti-His_6_ antibodies. As a control, non-induced *L. lactis* PA1001 pNG4110-*pro*-*atl* was included in the analysis (C). Pro-Atl is indicated with an *arrow*. **b** Purification of His_6_-Pro-Atl and His_6_-TEV-Pro-Atl from the supernatant of cells that were induced with nisin overnight. The LDS-PAGE shows (*1*) induced cells producing His_6_-Pro-Atl, (*2*) the culture supernatant fraction of cells producing His_6_-Pro-Atl, (*3*) the culture supernatant fraction of cells producing His_6_-TEV-Pro-Atl, (*4*/*5*) the first two elution fractions of His_6_-Pro-Atl upon metal affinity chromatography and (*6*/*7*) the first two elution fractions of His_6_-TEV-Pro-Atl upon metal affinity chromatography. The position of His_6_-tagged Pro-Atl is marked with an *arrow*, and the positions of Mw marker proteins are indicated

In a recent study, the N-terminal pro-peptide of the bifunctional autolysin Atl of *S. aureus* is reported to be recognized by antibodies in the plasma of patients with the genetic blistering disease epidermolysis bullosa (van den Berg et al. 2015). This intriguing finding initiated an effort to express the pro-Atl (amino acids 29–199) in *L. lactis* using the three vectors, pNG4110, pNG4111 and pNG4210. As shown in Fig. 6a, the N-terminally His_6_-tagged pro-Atl as expressed from pNG4110 and pNG4111 was efficiently produced and secreted (Fig. 6a). In fact, most of these His_6_-tagged forms of pro-Atl were detected in the growth medium from which they were readily purified by metal affinity chromatography (Fig. 6b). Compared to the N-terminally His_6_-tagged pro-Atl, only a minute amount of C-terminally His_6_-tagged pro-Atl as expressed from pNG4210 was detectable by Western blotting (Fig. 6a).

**Fig. 7:**
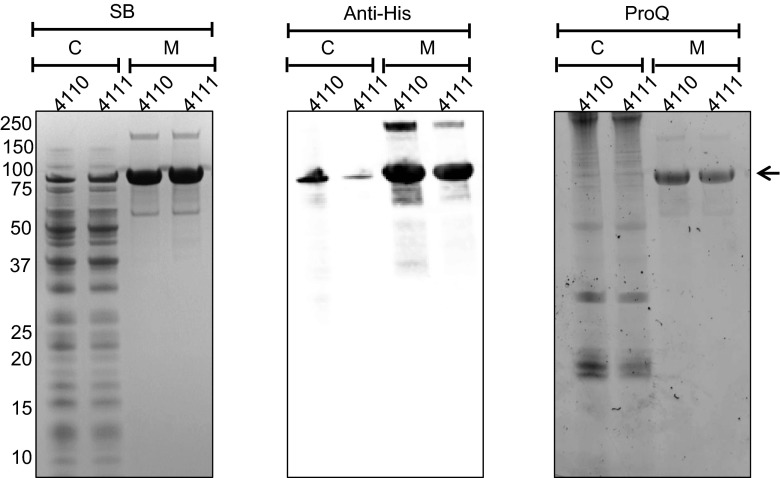
Extracellular production of phosphorylated *S. aureus* IsdB in *L. lactis*. **a** Cytosolic (C) and growth medium (M) fractions of *L. lactis* PA1001 producing His_6_-IsdB (4110) and His_6_-TEV-IsdB (4111) were analysed by LDS-PAGE and stained with SimplyBlue SafeStain (SB) or Pro-Q Diamond staining (Pro-Q). As a control, Western blotting was performed, using anti-His_6_ antibodies (α-His). The *arrow* marks the position of mature IsdB, and the positions of Mw marker proteins are indicated

### Secretion of phosphorylated IsdB by *L. lactis*

Recent studies by Basell et al. have shown that residues Tyr440 or Tyr444 of the covalently cell wall-attached IsdB protein of *S. aureus* are phosphorylated (Basell et al. 2014). To determine whether IsdB produced and secreted by *L. lactis* PA1001 is also phosphorylated, a 3′-truncated *isdB* gene lacking the sequences encoding the sortase recognition site was expressed from pNG4110 and pNG4111. As shown in Fig. 7, the N-terminally His_6_-tagged forms of IsdB were efficiently produced by *L. lactis*, and in fact, most of the protein is secreted into the growth medium. Importantly, phosphorylated His_6_-tagged IsdB was clearly detectable by gel staining with the Pro-Q® Diamond Phosphoprotein Gel Stain, showing *L. lactis* facilitates this post-translational modification. Whether the cytoplasmic forms of His_6_-IsdB and His_6_-TEV-IsdB as observed by Western blotting are also phosphorylated is presently unclear due to limited sensitivity of the Pro-Q staining in these extracts.

To pinpoint the site of IsdB phosphorylation in *L. lactis*, the secreted His_6_-TEV-IsdB was purified by metal affinity chromatography and applied to an LDS-PAA gel. The His_6_-TEV-IsdB band was subsequently excised from the gel, destained, washed and cleaved overnight with trypsin. Liberated peptides were analysed by MS/MS, which showed that His_6_-TEV-IsdB was phosphorylated on the Tyr311(^*^) residue in the peptide KYMVMETTNDDY^*^WKDFMVEGQR (Supplementary Fig. S1). The same Tyr residue was also shown to be phosphorylated when TCA-precipitated His_6_-TEV-IsdB from the culture supernatant of *L. lactis* PA1001 containing pNG4111-IsdB was used for the MS/MS analysis.

## Discussion

In the present study we describe a set of three cloning vectors for heterologous protein expression in *L. lactis*. These vectors enable easy exchange of the gene of interest for expression of protein variants with (i) a N-terminal His_6_-tag, (ii) a C-terminal His_6_-tag and (iii) a TEV protease cleavable C-terminal His_6_ protein. Examples of the successful use of the three vectors are presented for expression of six extracytoplasmic proteins from *S. aureus*, two of which were subsequently purified in one step by metal affinity chromatography.

Importantly, the position of the His_6_-tag purification label, either at the N- or the C-terminus, can have a major impact on the production level of *S. aureus* originated proteins expressed in *L. lactis*. This was particularly evident for SA0620, SA2100 and pro-Atl, which were only detectable when expressed as N-terminal His_6_-tagged protein. In contrast, no effect of the His_6_-tag position was observed for the production of FtsL, ClfB and IsdB (data not shown for FtsL and IsdB). The reason(s) for the negative impact of a C-terminal His_6_-tag on the production of SA0620, SA2100 and pro-Atl is presently not clear. For the SA0620 protein, the tag appears to interfere with the protein stability/folding as indicated by the presence of a degradation product. Similar degradation products are observed for the C-terminally His_6_-tagged ClfB, whereas these are not observed for the N-terminally His_6_-tagged ClfB. Similarly, the TEV cleavage site as encoded by pNG4111 may influence the protein production level. This was most evident for SA0620, although effects of the TEV cleavage site on the levels of detected product were also observed for ClfB and pro-Atl. However, for the latter two proteins, it is presently not entirely clear whether the TEV site interfered with protein production per se or with the immunodetection of the His_6_-tag with anti-His_6_-tag antibodies. The latter idea would be supported by the experiments on ClfB production, where there was a discrepancy between the ClfB levels detected by SimplyBlue gel staining and immunodetection.

With the exception of SA0620, all *S. aureus* proteins expressed in *L. lactis* were secreted into the growth medium with the help of the vector-encoded Usp45 signal peptide. The cause of the deviating behaviour of SA0620, which is part of the core exoproteome of *S. aureus* (Sibbald et al. 2006; Ziebandt et al. 2010), is unknown. Apparently, this protein has a particular unidentified feature that interferes with its secretion in *L. lactis*. This could, for example, relate to its targeting to the Sec secretion machinery, pre-translocational control of folding by chaperones or efficient post-translocational folding by dedicated folding catalysts that could be present in *S. aureus* but absent from *L. lactis*.(Sarvas et al. 2004; Bolhuis et al. 1999; Tjalsma et al. 2004)

The expression host strain used for the present studies was *L. lactis* PA1001, which lacks the major lactococcal proteases PrtP and HtrA (Poquet et al. 2000; Liu et al. 2010). Despite the absence of these two proteases, our results show that proteolysis of expressed proteins can still occur. This focuses attention on the remaining proteases that could be responsible for this unwanted effect. Conceivably, the responsible protease could be located in the cytoplasm (e.g. ClpP; Frees et al. 2001), the membrane (e.g. FtsH or a RseP-like protease; Dalbey et al. 2012) or an as yet unidentified protease in the cell wall. The elimination of this remnant protease activity could be beneficial for further production strain improvement, similar to what was shown for heterologous protein production in *B. subtilis* where the deletion of multiple protease genes resulted in large improvements in the production of heterologous proteins (Pohl et al. 2013; Krishnappa et al. 2014).

Lastly, our analysis of the production of *S. aureus* IsdB in *L. lactis* shows that this heterologous expression host does phosphorylate the IsdB protein as is the case in *S. aureus* (Basell et al. 2014). However, while in *S. aureus* either Tyr440 or Tyr444 are phosphorylated, the IsdB protein expressed in *L. lactis* was found to be phosphorylated on Tyr311. At present, the molecular basis for this apparently different choice of phosphorylation sites in *S. aureus* and *L. lactis* is not clear. One possible reason for the different results could be that the sample preparation was performed differently. While Basell et al. (2014) applied a gel-free proteomics analysis where phosphopeptides were enriched with TiO_2_, in the present analyses the IsdB protein was extracted from gel slices. Another perhaps more plausible reason could be that we expressed in *L. lactis* a 3′-truncated *isdB* gene lacking the sequences encoding the sortase recognition site and encoding a TEV protease cleavable N-terminal His_6_-tag. This may have resulted in an aberrant presentation of IsdB to the as yet unknown kinase responsible for phosphorylation of this protein. In contrast, the study in *S. aureus* addressed the authentic IsdB protein synthesized with the sortase cleavage site and, therefore, covalently anchored to the cell wall. A third possibility would be that the kinases responsible for IsdB phosphorylation in *S. aureus* and *L. lactis* have somewhat different specificities. A careful comparative analysis of the mechanisms of IsdB phosphorylation in *S. aureus* and *L. lactis* may pinpoint possible host-specific differences.

In conclusion, based on our present findings, we are confident that the newly developed vectors combined with the *L. lactis* expression host PA1001 have the potential to become very useful tools for the extracytoplasmic production and purification of bacterial antigens. Such a tool facilitates structural and functional studies on previously hard-to-produce proteins and may provide a starting point for vaccine development.

## Electronic supplementary material

ESM 1 (PDF 580 kb)

## References

[CR1] Arnau J, Lauritzen C, Petersen GE, Pedersen J (2011) Reprint of: Current strategies for the use of affinity tags and tag removal for the purification of recombinant proteins. Protein Expr Purif 10.1016/j.pep.2011.08.009 21889989

[CR2] Bardoel BW, Van Kessel KPM, Van Strijp JAG, Milder FJ (2012). Inhibition of *Pseudomonas aeruginosa* virulence: characterization of the AprA-AprI interface and species selectivity. J Mol Biol.

[CR3] Basell K, Otto A, Junker S, Zuhlke D, Rappen GM, Schmidt S, Hentschker C, Macek B, Ohlsen K, Hecker M, Becher D (2014). The phosphoproteome and its physiological dynamics in *Staphylococcus aureus*. Int J Med Microbiol.

[CR4] Bolhuis A, Tjalsma H, Smith HE, de Jong A, Meima R, Venema G, Bron S, van Dijl JM (1999). Evaluation of bottlenecks in the late stages of protein secretion in *Bacillus subtilis*. Appl Environ Microbiol.

[CR5] Bonn F, Bartel J, Buttner K, Hecker M, Otto A, Becher D (2014). Picking vanished proteins from the void: how to collect and ship/share extremely dilute proteins in a reproducible and highly efficient manner. Anal Chem.

[CR6] Bosma T, Kanninga R, Neef J, Audouy SAL, Van Roosmalen ML, Steen A, Buist G, Kok J, Kuipers OP, Robillard G, Leenhouts K (2006). Novel surface display system for proteins on non-genetically modified gram positive bacteria. Appl Environ Microbiol.

[CR7] Braun P, Gerritse G, van Dijl JM, Quax WJ (1999). Improving protein secretion by engineering components of the bacterial translocation machinery. Curr Opin Biotechnol.

[CR8] Buist G, Kok J, Leenhouts KJ, Dabrowska M, Venema G, Haandrikman AJ (1995). Molecular cloning and nucleotide sequence of the gene encoding the major peptidoglycan hydrolase of *Lactococcus lactis*, a muramidase needed for cell separation. J Bacteriol.

[CR9] Buist G, Karsens H, Nauta A, Van Sinderen D, Venema G, Kok J (1997). Autolysis of *Lactococcus lactis* caused by induced overproduction of its major autolysin, AcmA. Appl Environ Microbiol.

[CR10] Chen R (2012). Bacterial expression systems for recombinant protein production: *E. coli* and beyond. Biotechnol Adv.

[CR11] Cortes-Perez NG, Poquet I, Oliveria MN, Gratadoux JJ, Madsen SM, Miyoshi A, Corthier G, Azevedo V, Langella P, Bermudez-Humaran LG (2006). Construction and characterization of a *Lactococcus lactis* strain deficient in intracellular ClpP and extracellular HtrA proteases. Microbiology.

[CR12] Dalbey RE, Wang P, van Dijl JM (2012). Membrane proteases in the bacterial protein secretion and quality control pathway. Microbiol Mol Biol Rev.

[CR13] De Ruyter PGGA, Kuipers OP, De Vos WM (1996). Controlled gene expression systems for *Lactococcus lactis* with the food-grade inducer nisin. Appl Environ Microbiol.

[CR14] Duthie ES, Lorenz LL (1952). Staphylococcal coagulase; mode of action and antigenicity. J Gen Microbiol.

[CR15] Frees D, Varmanen P, Ingmer H (2001). Inactivation of a gene that is highly conserved in Gram-positive bacteria stimulates degradation of non-native proteins and concomitantly increases stress tolerance in *Lactococcus lactis*. Mol Microbiol.

[CR16] Jones C, Patel A, Griffin S, Martin J, Young P, O’Donnell K, Silverman C, Porter T, Chaiken I (1995). Current trends in molecular recognition and bioseparation. J Chromatogr A.

[CR17] Kreiswirth BN, Löfdahl S, Betley MJ, O’Reilly M, Schlievert PM, Bergdoll MS, Novick RP (1983). The toxic shock syndrome exotoxin structural gene is not detectably transmitted by a prophage. Nature.

[CR18] Krishnappa L, Dreisbach A, Otto A, Goosens VJ, Cranenburgh R, Harwood CR, Becher D, Van Dijl JM (2013). Extracytoplasmic proteases determining the cleavage and release of secreted proteins, lipoproteins, and membrane proteins in *Bacillus subtilis*. J Proteome Res.

[CR19] Krishnappa L, Monteferrante CG, Neef J, Dreisbach A, Van Dijl JM (2014). Degradation of extracytoplasmic catalysts for protein folding in *Bacillus subtilis*. Appl Environ Microbiol.

[CR20] Kuipers OP, Beerthuyzen MM, Siezen RJ, De Vos WM (1993). Characterization of the nisin gene cluster *nisABTCIPR* of *Lactococcus lactis*. Requirement of expression of the *nisA* and *nisI* genes for development of immunity. Eur J Biochem.

[CR21] Kuipers OP, De Ruyter PGGA, Kleerebezem M, De Vos WM (1997). Controlled overproduction of proteins by lactic acid bacteria. Trends Biotechnol.

[CR22] Kuroda M, Ohta T, Uchiyama I, Baba T, Yuzawa H, Kobayashi I, Cui L, Oguchi A, Aoki K, Nagai Y, Lian J, Ito T, Kanamori M, Matsumaru H, Maruyama A, Murakami H, Hosoyama A, Mizutani-Ui Y, Takahashi NK, Sawano T, Inoue R, Kaito C, Sekimizu K, Hirakawa H, Kuhara S, Goto S, Yabuzaki J, Kanehisa M, Yamashita A, Oshima K, Furuya K, Yoshino C, Shiba T, Hattori M, Ogasawara N, Hayashi H, Hiramatsu K (2001). Whole genome sequencing of meticillin-resistant *Staphylococcus aureus*. Lancet.

[CR23] Leenhouts KJ, Venema G, Hardy KG (1993). Lactococcal plasmid vectors. Plasmids, a practical approach.

[CR24] Li W, Zhou X, Lu P (2004). Bottlenecks in the expression and secretion of heterologous proteins in *Bacillus subtilis*. Res Microbiol.

[CR25] Liu M, Bayjanov JR, Renckens B, Nauta A, Siezen RJ (2010). The proteolytic system of lactic acid bacteria revisited: a genomic comparison. BMC Genomics.

[CR26] McDougal LK, Steward CD, Killgore GE, Chaitram JM, McAllister SK, Tenover FC (2003). Pulsed-field gel electrophoresis typing of oxacillin-resistant *Staphylococcus aureus* isolates from the United States: establishing a national database. J Clin Microbiol.

[CR27] Mierau IKM (2005). 10 Years of the nisin controlled gene expression system (NICE) in *Lactococcus lactis*. Appl Microbiol Biotechnol.

[CR28] Miyoshi A, Poquet I, Azevedo V, Commissaire J, Bermudez-Humaran LG, Domakova E, Le Loir Y, Oliveira SC, Grusse A, Langella P (2002). Controlled production of stable heterologous proteins in *Lactococcus lactis*. Appl Environ Microbiol.

[CR29] Morello E, Bermudez-Humaran LG, Llull D, Sole V, Miraglio N, Langella P, Poquet I (2008). *Lactococcus lactis*, an efficient cell factory for recombinant protein production and secretion. J Mol Microbiol Biotechnol.

[CR30] Neef J, Koedijk DG, Bosma T, van Dijl JM, Buist G (2014). Efficient production of secreted staphylococcal antigens in a non-lysing and proteolytically reduced *Lactococcus lactis* strain. Appl Microbiol Biotechnol.

[CR31] Pel MJ, van Dijken AJ, Bardoel BW, Seidl MF, van der Ent S, van Strijp JA, Pieterse CM (2014). *Pseudomonas syringae* evades host immunity by degrading flagellin monomers with alkaline protease AprA. Mol Plant Microbe Interact.

[CR32] Petsch D, Anspach FB (2000). Endotoxin removal from protein solutions. J Biotechnol.

[CR33] Pohl S, Bhavsar G, Hulme J, Bloor AE, Misirli G, Leckenby MW, Radford DS, Smith W, Wipat A, Williamson ED, Harwood CR, Cranenburgh RM (2013). Proteomic analysis of *Bacillus subtilis* strains engineered for improved production of heterologous proteins. Proteomics.

[CR34] Poquet I, Saint V, Seznec E, Simoes N, Bolotin A, Gruss A (2000). HtrA is the unique surface housekeeping protease in *Lactococcus lactis* and is required for natural protein processing. Mol Microbiol.

[CR35] Sarvas M, Harwood CR, Bron S, Van Dijl JM (2004). Post-translocational folding of secretory proteins in Gram-positive bacteria. Biochim Biophys Acta.

[CR36] Sibbald MJJB, Ziebandt AK, Engelmann S, Hecker M, De Jong A, Hamsen HJM, Raangs GC, Stokroos I, Arends JP, Dubois JYF, Van Dijl JM (2006). Mapping the pathway to staphylococcal pathogenesis by comparative secretomics. Microbiol Mol Biol Rev.

[CR37] Terpe K (2003). Overview of tag protein fusions: from molecular and biochemical fundamentals to commercial systems. Appl Microbiol Biotechnol.

[CR38] Terpe K (2006). Overview of bacterial expression system for heterologous protein production from molecular and biochemical fundamentals to commercial systems. Appl Microbiol Biotechnol.

[CR39] Tjalsma H, Koetje EJ, Kiewiet R, Kuipers OP, Kolkman M, Van der Laan J, Daskin R, Ferrari E, Bron S (2004). Engineering of quorum-sensing systems for improved production of alkaline protease by *Bacillus subtilis*. J Appl Microbiol.

[CR40] van den Berg S, Koedijk DG, Back JW, Neef J, Dreisbach A, van Dijl JM, Bakker-Woudenberg IA, Buist G (2015). Active immunization with an octa-valent *Staphylococcus aureus* antigen mixture in models of *S. aureus* bacteremia and skin infection in mice. PLoS One.

[CR41] Westers L, Westers H, Quax WJ (2004). *Bacillus subtilis* as cell factory for pharmaceutical proteins: a biotechnological approach to optimize the host organism. Biochim Biophys Acta.

[CR42] Woestenenk EA, Hammarstrom M, van den Berg S, Hard T, Berglund H (2004). His tag effect on solubility of human proteins produced in *Escherichia coli*: a comparison between four expression vectors. J Struct Funct Genomics.

[CR43] Zerbs S, Frank AM, Collart FR (2009). Bacterial systems for production of heterologous proteins. Methods Enzymol.

[CR44] Zhou XX, Li WF, Ma GX, Pan YJ (2006). The nisin-controlled gene expression system: construction, application and improvements. Biotechnol Adv.

[CR45] Ziebandt AK, Kusch H, Degner M, Jaglitz S, Sibbald MJ, Arends JP, Chlebowicz MA, Albrecht D, Pantucek R, Doskar J, Ziebuhr W, Broker BM, Hecker M, Van Dijl JM, Engelmann S (2010). Proteomics uncovers extreme heterogeneity in the *Staphylococcus aureus* exoproteome due to genomic plasticity and variant gene regulation. Proteomics.

