# Supplementary material for: Versatile vector suite for the extracytoplasmic production and purification of heterologous His-tagged proteins in Lactococcus lactis
Source: Appl Microbiol Biotechnol. 2015 Jul 10;99(21):9037–48. doi: 10.1007/s00253-015-6778-8 (PMC4619460; doi:10.1007/s00253-015-6778-8)
Supplement: Supplementary file 1 — (PDF 580 kb) [file 253_2015_6778_MOESM1_ESM.pdf]

Supplementary material submitted to "Applied Microbiology and Biotechnology" for:

Versatile vector suite for the extracytoplasmic production and purification of heterologous His-tagged proteins in *Lactococcus lactis*

Jolanda Neef[1], Fin J. Milder[2], Danny G.A.M. Koedijk[1], Marindy Klaassens[1], Erik C. Heezius[2], Jos A.G. van Strijp[2], Andreas Otto[3], Dörte Becher[3], Jan Maarten van Dijl[1], and Girbe Buist[1#]

[1] Department of Medical Microbiology, University of Groningen, University Medical Center Gronigen, Hanzeplein 1, P.O. Box 30001, 9700 RB Groningen, The Netherlands

[2] Department of Medical Microbiology, University Medical Center Utrecht, PO G04.614, Heidelberglaan 100, 3584 CX Utrecht, The Netherlands

[3]Institut für Mikrobiologie, Ernst-Moritz-Arndt Universität Greifswald, Friedrich-Ludwig-Jahn-Str. 15, D-17489 Greifswald, Germany

[#]**Corresponding author**: Girbe Buist, Department of Medical Microbiology, University Medical Center Groningen, Hanzeplein 1, P.O. Box 30001, 9700 RB Groningen, the Netherlands. Phone: +31-50-3615190; Fax: +31-50-3619105; E-mail: g.buist@umcg.nl

Table S1: Primers used in this study

Primer	5'→ 3'Nucleotide sequence[a]	Restriction site
SA0620.f	ATATGGATCCATGGGATCTACACAACATACAGTACAATCTGG	*Bam*HI
SA0620.r1	ATATGCGGCCGCTCGAATTCCATGGATGAATGCATAGC	*Not*I
SA0620.r2	ATATGCGGCCGC**TTA**TCGAATTCCATGGATGAATGCATAGC	*Not*I
FtsL.f	ATATGGATCCAAAATGGATGCGTATGATACGCG	*Bam*HI
FtsL.r1	ATATGCGGCCGCATTTTTTGCTTCGCCATTACTAC	*Not*I
FtsL.r2	ATATGCGGCCGC**TTA**ATTTTTTGCTTCGCCATTACTACGC	*Not*I
ClfB.f	ATATGGATCCTCAGAACAATCGAACGATACAACGC	*Bam*HI
ClfB.r1	ATATGCGGCCGCGTCTGGGCTTGGTTCCGGTTC	*Not*I
ClfB.r2	ATATGCGGCCGC**TTA**GTCTGGGCTTGGTTCCGGTTC	*Not*I
SA2100.f	ATATGGATCCTCTGAGCAAGATAACTACGGTTATAATCC	*Bam*HI
SA2100.r1	ATATGCGGCCGCGTGAATGAAGTTATAACCAGCAGCTTGG	*Not*I
SA2100.r2	ATATGCGGCCGC**TTA**GTGAATGAAGTTATAACCAGCAGCTTGG	*Not*I
Pro-Atl.f	ATATGGATCCGCTGAGACGACACAAGATCAAACTACTAATAAAAACG	*Bam*HI
Pro-Atl.r1	ATATGCGGCCGCAGCGCTAAAAGTAGTTACTTTAGGTGTCGCTTCAGTTTTAGC	*Not*I
Pro-Atl.r2	ATATGCGGCCGC**TTA**AGCGCTAAAAGTAGTTACTTTAGGTGTCGCTTCAGTTTTAGC	*Not*I
IsdB.f	ATATGGATCCGCAGCTGAAGAAACAGGTGGTACAAATAC	*Bam*HI
IsdB.r1	ATATGCGGCCGCTGATTTTGCTTTATTTTCTTGTGTATTTTTATTG	*Not*I
IsdB.r2	ATATGCGGCCGC**TTA**TGATTTTGCTTTATTTTCTTGTGTATTTTTATTG	*Not*I

[a]Restriction sites underlined, stop codons in bold

Figure S1: MS/MS results to identify the phosphorylation site of purified (a-c) and TCA precipitated (d) His$_6$-TEV-IsdB.

a

(K)KYMVMETTNDDyWKDFmVEGQR(V)

purified His$_6$-TEV-IsdB

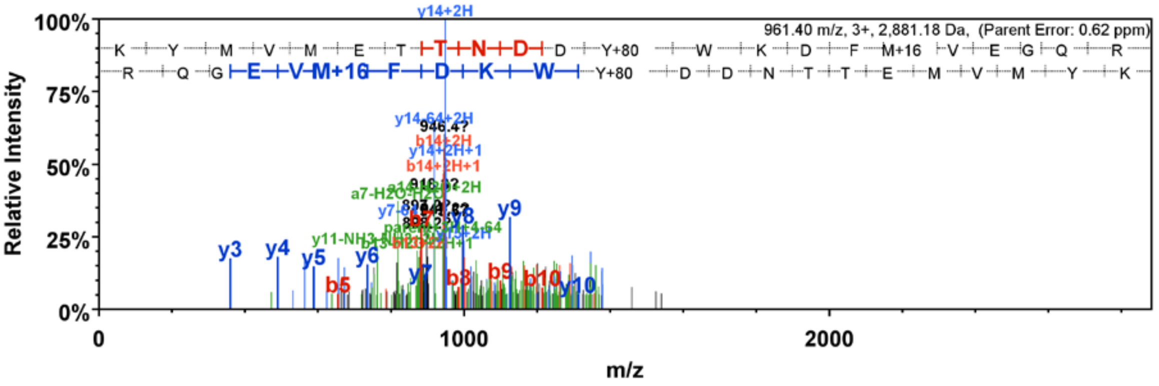

B	B Ions	B+2H	B-NH3	B-H2O	AA	Y Ions	Y+2H	Y-NH3	Y-H2O	Y
1	129.1	65.1	112.1		K	2,882.2	1,441.6	2,865.2	2,864.2	22
2	292.2	146.6	275.1	274.2	Y	2,754.1	1,377.5	2,737.1	2,736.1	21
3	423.2	212.1	406.2	405.2	M	2,591.0	1,296.0	2,574.0	2,573.0	20
4	522.3	261.6	505.2	504.3	V	2,460.0	1,230.5	2,443.0	2,442.0	19
5	653.3	327.2	636.3	635.3	M	2,360.9	1,181.0	2,343.9	2,342.9	18
6	782.4	391.7	765.3	764.3	E	2,229.9	1,115.4	2,212.9	2,211.9	17
7	883.4	442.2	866.4	865.4	T	2,100.8	1,050.9	2,083.8	2,082.8	16
8	984.5	492.7	967.4	966.4	T	1,999.8	1,000.4	1,982.8	1,981.8	15
9	1,098.5	549.8	1,081.5	1,080.5	N	1,898.7	949.9	1,881.7	1,880.7	14
10	1,213.5	607.3	1,196.5	1,195.5	D	1,784.7	892.9	1,767.7	1,766.7	13
11	1,328.5	664.8	1,311.5	1,310.5	D	1,669.7	835.3	1,652.6	1,651.7	12
12	1,571.6	786.3	1,554.6	1,553.6	Y+80	1,554.6	777.8	1,537.6	1,536.6	11
13	1,757.7	879.3	1,740.6	1,739.6	W	1,311.6	656.3	1,294.6	1,293.6	10
14	1,885.8	943.4	1,868.7	1,867.7	K	1,125.5	563.3	1,108.5	1,107.5	9
15	2,000.8	1,000.9	1,983.8	1,982.8	D	997.4	499.2	980.4	979.4	8
16	2,147.8	1,074.4	2,130.8	2,129.8	F	882.4	441.7	865.4	864.4	7
17	2,294.9	1,147.9	2,277.9	2,276.9	M+16	735.3	368.2	718.3	717.3	6
18	2,394.0	1,197.5	2,376.9	2,375.9	V	588.3	294.7	571.3	570.3	5
19	2,523.0	1,262.0	2,506.0	2,505.0	E	489.2	245.1	472.2	471.2	4
20	2,580.0	1,290.5	2,563.0	2,562.0	G	360.2	180.6	343.2	342.2	3
21	2,708.1	1,354.5	2,691.0	2,690.1	Q	303.2	152.1	286.2	285.2	2
22	2,882.2	1,441.6	2,865.2	2,864.2	R	175.1	88.1	158.1		1

Xcorr	DCn	Actual peptide mass (AMU)	Spectrum charge	Δmass (PPM)
4.05	0.354	2,881.18	3	0.62

b

(K)KYMVMETTNDDyWKDFmVEGQR(V)

purified His$_6$-TEV-IsdB

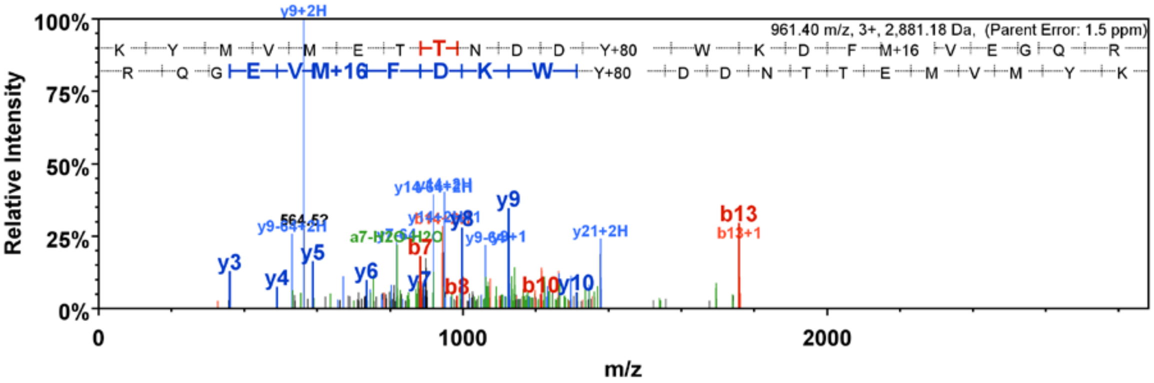

B	B Ions	B+2H	B-NH3	B-H2O	AA	Y Ions	Y+2H	Y-NH3	Y-H2O	Y
1	129.1	65.1	112.1		K	2,882.2	1,441.6	2,865.2	2,864.2	22
2	292.2	146.6	275.1	274.2	Y	2,754.1	1,377.5	2,737.1	2,736.1	21
3	423.2	212.1	406.2	405.2	M	2,591.0	1,296.0	2,574.0	2,573.0	20
4	522.3	261.6	505.2	504.3	V	2,460.0	1,230.5	2,443.0	2,442.0	19
5	653.3	327.2	636.3	635.3	M	2,360.9	1,181.0	2,343.9	2,342.9	18
6	782.4	391.7	765.3	764.3	E	2,229.9	1,115.4	2,212.9	2,211.9	17
7	883.4	442.2	866.4	865.4	T	2,100.8	1,050.9	2,083.8	2,082.8	16
8	984.5	492.7	967.4	966.4	T	1,999.8	1,000.4	1,982.8	1,981.8	15
9	1,098.5	549.8	1,081.5	1,080.5	N	1,898.7	949.9	1,881.7	1,880.7	14
10	1,213.5	607.3	1,196.5	1,195.5	D	1,784.7	892.9	1,767.7	1,766.7	13
11	1,328.5	664.8	1,311.5	1,310.5	D	1,669.7	835.3	1,652.6	1,651.7	12
12	1,571.6	786.3	1,554.6	1,553.6	Y+80	1,554.6	777.8	1,537.6	1,536.6	11
13	1,757.7	879.3	1,740.6	1,739.6	W	1,311.6	656.3	1,294.6	1,293.6	10
14	1,885.8	943.4	1,868.7	1,867.7	K	1,125.5	563.3	1,108.5	1,107.5	9
15	2,000.8	1,000.9	1,983.8	1,982.8	D	997.4	499.2	980.4	979.4	8
16	2,147.8	1,074.4	2,130.8	2,129.8	F	882.4	441.7	865.4	864.4	7
17	2,294.9	1,147.9	2,277.9	2,276.9	M+16	735.3	368.2	718.3	717.3	6
18	2,394.0	1,197.5	2,376.9	2,375.9	V	588.3	294.7	571.3	570.3	5
19	2,523.0	1,262.0	2,506.0	2,505.0	E	489.2	245.1	472.2	471.2	4
20	2,580.0	1,290.5	2,563.0	2,562.0	G	360.2	180.6	343.2	342.2	3
21	2,708.1	1,354.5	2,691.0	2,690.1	Q	303.2	152.1	286.2	285.2	2
22	2,882.2	1,441.6	2,865.2	2,864.2	R	175.1	88.1	158.1		1

Xcorr	DCn	Actual peptide mass (AMU)	Spectrum charge	Δmass (PPM)
3.66	0.434	2,881.18	3	1.5

c

(K)KYMVMETTNDDyWKDFmVEGQR(V)

purified His$_6$-TEV-IsdB

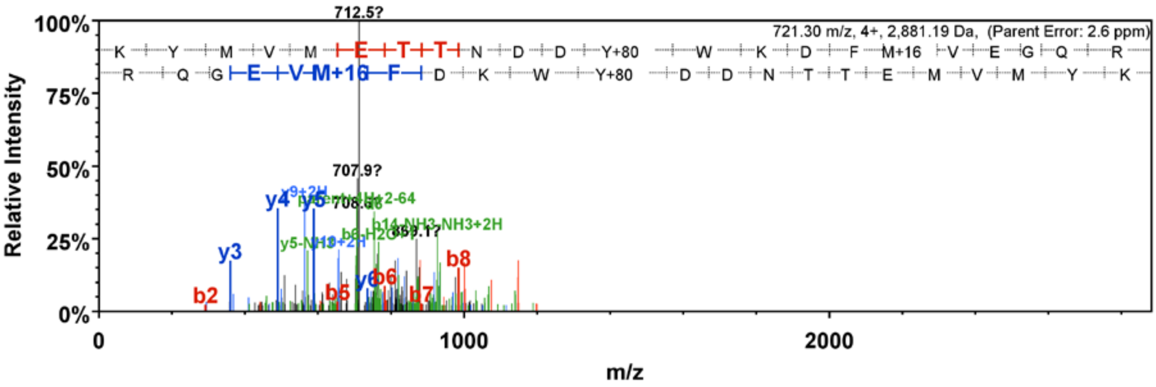

B	B Ions	B+2H	B-NH3	B-H2O	AA	Y Ions	Y+2H	Y-NH3	Y-H2O	Y
1	129.1	65.1	112.1		K	2,882.2	1,441.6	2,865.2	2,864.2	22
2	292.2	146.6	275.1	274.2	Y	2,754.1	1,377.5	2,737.1	2,736.1	21
3	423.2	212.1	406.2	405.2	M	2,591.0	1,296.0	2,574.0	2,573.0	20
4	522.3	261.6	505.2	504.3	V	2,460.0	1,230.5	2,443.0	2,442.0	19
5	653.3	327.2	636.3	635.3	M	2,360.9	1,181.0	2,343.9	2,342.9	18
6	782.4	391.7	765.3	764.3	E	2,229.9	1,115.4	2,212.9	2,211.9	17
7	883.4	442.2	866.4	865.4	T	2,100.8	1,050.9	2,083.8	2,082.8	16
8	984.5	492.7	967.4	966.4	T	1,999.8	1,000.4	1,982.8	1,981.8	15
9	1,098.5	549.8	1,081.5	1,080.5	N	1,898.7	949.9	1,881.7	1,880.7	14
10	1,213.5	607.3	1,196.5	1,195.5	D	1,784.7	892.9	1,767.7	1,766.7	13
11	1,328.5	664.8	1,311.5	1,310.5	D	1,669.7	835.3	1,652.6	1,651.7	12
12	1,571.6	786.3	1,554.6	1,553.6	Y+80	1,554.6	777.8	1,537.6	1,536.6	11
13	1,757.7	879.3	1,740.6	1,739.6	W	1,311.6	656.3	1,294.6	1,293.6	10
14	1,885.8	943.4	1,868.7	1,867.7	K	1,125.5	563.3	1,108.5	1,107.5	9
15	2,000.8	1,000.9	1,983.8	1,982.8	D	997.4	499.2	980.4	979.4	8
16	2,147.8	1,074.4	2,130.8	2,129.8	F	882.4	441.7	865.4	864.4	7
17	2,294.9	1,147.9	2,277.9	2,276.9	M+16	735.3	368.2	718.3	717.3	6
18	2,394.0	1,197.5	2,376.9	2,375.9	V	588.3	294.7	571.3	570.3	5
19	2,523.0	1,262.0	2,506.0	2,505.0	E	489.2	245.1	472.2	471.2	4
20	2,580.0	1,290.5	2,563.0	2,562.0	G	360.2	180.6	343.2	342.2	3
21	2,708.1	1,354.5	2,691.0	2,690.1	Q	303.2	152.1	286.2	285.2	2
22	2,882.2	1,441.6	2,865.2	2,864.2	R	175.1	88.1	158.1		1

Xcorr	DCn	Actual peptide mass (AMU)	Spectrum charge	Δmass (PPM)
3.55	0.223	2,881.19	4	2.6

d

(K)KYMVMETTNDDyWKDFmVEGQR(V)

TCA-precipitated His$_6$-TEV-IsdB

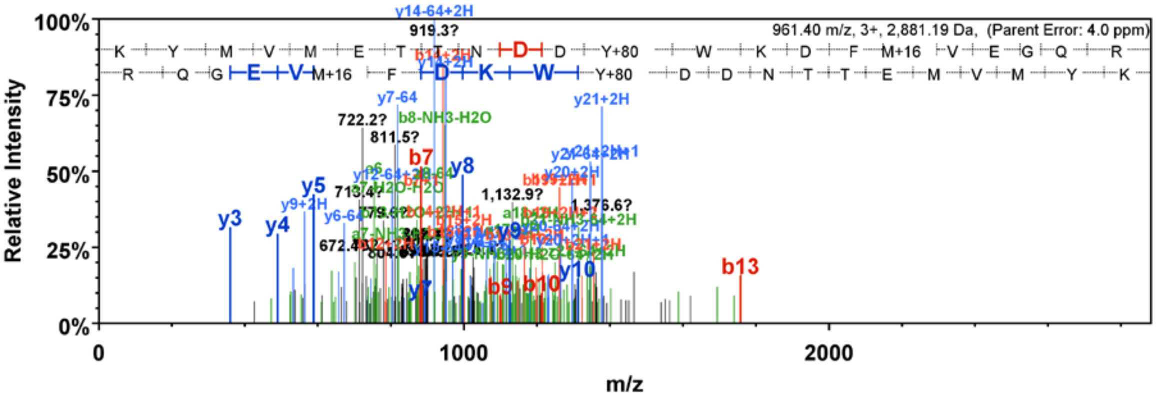

B	B Ions	B+2H	B-NH3	B-H2O	AA	Y Ions	Y+2H	Y-NH3	Y-H2O	Y
1	129.1	65.1	112.1		K	2,882.2	1,441.6	2,865.2	2,864.2	22
2	292.2	146.6	275.1	274.2	Y	2,754.1	1,377.5	2,737.1	2,736.1	21
3	423.2	212.1	406.2	405.2	M	2,591.0	1,296.0	2,574.0	2,573.0	20
4	522.3	261.6	505.2	504.3	V	2,460.0	1,230.5	2,443.0	2,442.0	19
5	653.3	327.2	636.3	635.3	M	2,360.9	1,181.0	2,343.9	2,342.9	18
6	782.4	391.7	765.3	764.3	E	2,229.9	1,115.4	2,212.9	2,211.9	17
7	883.4	442.2	866.4	865.4	T	2,100.8	1,050.9	2,083.8	2,082.8	16
8	984.5	492.7	967.4	966.4	T	1,999.8	1,000.4	1,982.8	1,981.8	15
9	1,098.5	549.8	1,081.5	1,080.5	N	1,898.7	949.9	1,881.7	1,880.7	14
10	1,213.5	607.3	1,196.5	1,195.5	D	1,784.7	892.9	1,767.7	1,766.7	13
11	1,328.5	664.8	1,311.5	1,310.5	D	1,669.7	835.3	1,652.6	1,651.7	12
12	1,571.6	786.3	1,554.6	1,553.6	Y+80	1,554.6	777.8	1,537.6	1,536.6	11
13	1,757.7	879.3	1,740.6	1,739.6	W	1,311.6	656.3	1,294.6	1,293.6	10
14	1,885.8	943.4	1,868.7	1,867.7	K	1,125.5	563.3	1,108.5	1,107.5	9
15	2,000.8	1,000.9	1,983.8	1,982.8	D	997.4	499.2	980.4	979.4	8
16	2,147.8	1,074.4	2,130.8	2,129.8	F	882.4	441.7	865.4	864.4	7
17	2,294.9	1,147.9	2,277.9	2,276.9	M+16	735.3	368.2	718.3	717.3	6
18	2,394.0	1,197.5	2,376.9	2,375.9	V	588.3	294.7	571.3	570.3	5
19	2,523.0	1,262.0	2,506.0	2,505.0	E	489.2	245.1	472.2	471.2	4
20	2,580.0	1,290.5	2,563.0	2,562.0	G	360.2	180.6	343.2	342.2	3
21	2,708.1	1,354.5	2,691.0	2,690.1	Q	303.2	152.1	286.2	285.2	2
22	2,882.2	1,441.6	2,865.2	2,864.2	R	175.1	88.1	158.1		1

Xcorr	DCn	Actual peptide mass (AMU)	Spectrum charge	Δmass (PPM)
3.87	0.543	2,881.19	3	4
